# The Spectroscope

**Published:** 1870-02

**Authors:** 


THE SPECTROSCOPE.—The science of solar and stellar chemistry is in its infancy. Its birth is within the memory of the boy just beginning the study of the physical sciences. And yet, how varied are its applications! how measureless the scope of its achievements! It has discovered new elements, which exist in such minute quantities, that though very widely diffused, they were never detected by the most delicate methods of analysis before known to chemists. It can detect the 1-6,000,000th of a grain of lithium, or the 1-180,000,000th of a grain of sodium, in a compound. It can take the wings of light and visit the sun, millions of miles away from our little earth, can analyze the fiery ocean of his atmosphere, and tell us what metals, vaporized by the fervent heat, are mingled in those burning billows. It flies away to stars so distant, that light, traveling at more than ten millions of miles a minute, takes ten, twenty, fifty, or even hundreds of *years* to accomplish the journey; it analyzes the atmosphere of those remote suns, and selects the metals and gasses that are floating therein Suns, so far off that through the best telescope they appear only as glittering points, are as truly in the hands of the chemist as the substances he melts in his crucibles. Compared with this, even the boasted miracles of modern science sink to insignificance. The annihilation of time and space by the electric telegraph, speeding across continents and beneath oceans, is as nothing to this immeasurable sweep of spectral analysis which overleaps the abyss between world and world, system and system.—*Boston Journal of Chemistry.*

